# Sparsentan for Focal Segmental Glomerulosclerosis in the DUET Open-Label Extension: Long-term Efficacy and Safety

**DOI:** 10.1016/j.xkme.2024.100833

**Published:** 2024-04-26

**Authors:** Kirk N. Campbell, Loreto Gesualdo, Edward Murphy, Michelle N. Rheault, Tarak Srivastava, Vladimir Tesar, Radko Komers, Howard Trachtman

**Affiliations:** 1 Icahn School of Medicine at Mount Sinai, New York, NY; 2 University of Bari Aldo Moro, Bari, Italy; 3 Travere Therapeutics, Inc, San Diego, CA; 4 University of Minnesota Medical School, Minneapolis, MN; 5 Children’s Mercy Hospital, Kansas City, MO; 6 Charles University, General University Hospital, Prague, Czech Republic; 7 University of Michigan, Ann Arbor, MI

**Keywords:** eGFR slope, FSGS partial remission endpoint, FPRE, kidney function, open-label extension, proteinuria, randomized controlled clinical trial, sparsentan

## Abstract

**Rationale & Objective:**

Sparsentan is a novel, non-immunosuppressive, single-molecule, dual endothelin angiotensin receptor antagonist (DEARA) examined in the ongoing phase 2 DUET trial for focal segmental glomerulosclerosis (FSGS). In the DUET 8-week double-blind period, sparsentan resulted in greater proteinuria reduction versus irbesartan. We report the long-term efficacy and safety of sparsentan during the open-label extension over more than 4 years.

**Study Design:**

Patients were examined from their first sparsentan dose (double-blind period or open-label extension) through 4.6 years.

**Setting & Participants:**

Patients with FSGS, excluding secondary FSGS.

**Intervention:**

Sparsentan (200, 400, and 800 mg/d).

**Outcomes:**

Urinary protein-creatinine ratio, FSGS partial remission endpoint (urinary protein-creatinine ratio ≤1.5 g/g and >40% reduction from baseline), estimated glomerular filtration rate, and blood pressure approximately every 12 weeks. Treatment-emergent adverse events by year and cases/100 patient-years.

**Results:**

109 patients were enrolled; 108 received ≥1 sparsentan dose; 103 entered the open-label extension (68 sparsentan, 35 irbesartan during the double-blind period). Sparsentan was ongoing in 45/108 patients (41.7%); median time to treatment discontinuation was 3.9 years (95% CI, 2.6-5.2). Mean percent proteinuria reduction from baseline was sustained through follow-up. Achieving partial remission within 9 months of first sparsentan dose (52.8% of patients) versus not achieving (47.2%) was associated with significantly slower rate of estimated glomerular filtration rate decline over the entire treatment period (−2.70 vs −6.56; *P* = 0.03) and in the first 2 years (−1.69 vs −6.46; *P* = 0.03). The most common treatment-emergent adverse events (>9 cases/100 patient-years) were headache, peripheral edema, upper respiratory infection, hyperkalemia, and hypotension. Peripheral edema and hypotension declined from year 1 (13.9% and 15.7% of patients, respectively) to ≤4% in years ≥2. There were no cases of heart failure and no patient deaths.

**Limitations:**

The open-label extension does not include a comparison group.

**Conclusions:**

Long-term sparsentan treatment showed sustained proteinuria reduction and a consistent safety profile.

## Plain-Language Summary

There is substantial unmet clinical need for safe and effective treatments for focal segmental glomerulosclerosis (FSGS), a kidney lesion with varied causes. Sparsentan is being studied for treatment of FSGS and targets 2 important pathways (endothelin-1 and angiotensin II) that lead to the loss of kidney function. In the 8-week randomized, double-blind DUET study in patients with FSGS, sparsentan reduced the amount of protein in the urine better than irbesartan (a blood pressure medicine often used to treat FSGS). We examined long-term treatment with sparsentan over >4 years in the DUET open-label extension. We found sustained proteinuria reduction in patients who continued treatment with sparsentan and a consistent safety profile with no new or unexpected adverse effects.

Focal segmental glomerulosclerosis (FSGS) is a histopathological kidney lesion resulting from initial injury to the podocytes that is characterized by segmental accumulation of extracellular matrix, resulting in capillary obliteration and glomerular scarring.^1^^,^^2^ Patients with FSGS present with a variable degree of proteinuria and often clinically evident nephrotic syndrome.^1^ FSGS accounts for a substantial proportion of adult (5%) and pediatric (12%) cases of kidney failure (KF).^3^^,^^4^ Frequent recurrence of FSGS, described in up to 55% of patients who receive a kidney transplant,^5^, ^6^, ^7^ represents another challenge for management and clinical research. Multiple causes lead to FSGS lesions, which can be categorized as primary,^8^^,^^9^ genetic,^10^^,^^11^ secondary,^1^^,^^9^ and unknown etiology.^12^

Most patients with FSGS regardless of etiology are routinely treated with renin-angiotensin-aldosterone system inhibitors (RAASis). Immunosuppressive therapy (IST) is generally reserved for patients with primary FSGS or nephrotic syndrome.^12^^,^^13^ However, these agents have limited efficacy and significant toxicity. There are no pharmacological therapies approved by the US Food and Drug Administration specifically for FSGS. Thus, a substantial unmet clinical need for safe and effective treatments exists.

In parallel with a variety of beneficial nephroprotective effects of RAASi, selective endothelin type A receptor (ET_A_R) antagonists have been shown to have anti-inflammatory, antifibrotic, antiproliferative, podocyte-protective, and hemodynamic effects in models of kidney diseases.^14^^,^^15^ Endothelin-1 and angiotensin II are key mediators of damage in FSGS and act in tandem through ET_A_R and the angiotensin II subtype 1 receptor (AT_1_R) to drive podocyte damage, inflammation, and sclerosis.^16^ The actions of RAASi and ET_A_R antagonists in combination have demonstrated additive benefits in experimental models of kidney disease^16^, ^17^, ^18^ and in patients with both diabetic^19^^,^^20^ and nondiabetic chronic kidney disease.^21^

Sparsentan is a novel, non-immunosuppressive, single-molecule dual endothelin angiotensin receptor antagonist with high selectivity for ET_A_R and AT_1_R.^17^ In the ongoing phase 2 DUET clinical trial in patients with FSGS, administration of sparsentan in the 8-week double-blind period achieved greater proteinuria reduction compared with active control irbesartan.^22^ After completion of the double-blind period, patients were offered the opportunity to participate in an open-label extension. Despite the single-arm design in the open-label extension, the DUET study and its open-label extension, initiated in 2014, provide a unique opportunity to assess long-term safety and several aspects of efficacy of sparsentan in altering the clinical course in patients with FSGS. In this post hoc analysis of the DUET open-label extension including almost 7 years of treatment, we pooled data from all patients who received sparsentan regardless of original randomization during the double-blind period. We report the long-term effects of sparsentan on proteinuria, blood pressure (BP), and preservation of kidney function, as well as the incidence and time course of adverse events (AEs).

## Methods

### Study Design and Treatment

A complete description of the DUET study design has been published.^22^^,^^23^ In brief, DUET is a double-blind, randomized, active-controlled, dose-escalation study followed by an open-label extension in patients with biopsy-proven FSGS or documentation of a pathogenic genetic mutation in a podocyte protein associated with the lesion.^22^ Secondary causes of FSGS were excluded based on the clinical evaluation performed by the site investigator. DUET enrolled patients at 44 sites between April 2014 and April 2016 in the United States and Europe, and the first patient entered the open-label extension in June 2014. The study was approved by the institutional review board or ethics committee at each participating site, in accordance with the Declaration of Helsinki. Eligible patients were 8-75 years old in the United States and 18-75 years old in Europe, with baseline urinary protein-creatinine ratio (UPCR) ≥1.0 g/g and estimated glomerular filtration rate (eGFR) >30 mL/min/1.73 m^2^.^22^ At baseline, chronic IST, except cyclophosphamide and rituximab, was permitted if dosing was stable for 1 month before randomization, and the dose was unchanged except for safety reasons during the double-blind period.

Enrolled patients taking an angiotensin receptor blocker and/or angiotensin-converting enzyme inhibitor underwent a 2-week washout period before day 1/randomization. Patients were initially assigned to a dose cohort and within each cohort randomized to receive either sparsentan or the active control (irbesartan 300 mg/d) for 8 weeks. Patients randomized to sparsentan received either 200, 400, or 800 mg/d. Adjustments in the drug doses were made for patients weighing ≤50 kg (ie, one-half the assigned dose).

Patients who completed the 8-week double-blind period were invited to enter the open-label extension and receive sparsentan. During the open-label extension, patients who initially received sparsentan continued with the same dose as at the last day of the double-blind period. In patients originally randomized to irbesartan, baseline for the current analysis was defined as day 56 (week 8), when they transitioned from irbesartan to sparsentan at the start of the open-label extension without an irbesartan washout. These patients received the dose of sparsentan corresponding to their original dose cohort allocation. Dose increases of sparsentan up to 800 mg/d for efficacy were allowed in the open-label extension. Similarly, dose reductions for safety or tolerability reasons were permitted during the open-label extension. Initiation of new IST or changes in ongoing IST were also allowed during the open-label extension at investigator discretion.

### Assessments and Procedures

Assessments were performed approximately every 12 weeks during the open-label extension, including BP, concomitant medications, and eGFR and proteinuria. The DUET open-label extension procedures were the same as during the double-blind period.^22^^,^^23^ Peripheral blood and urine samples were analyzed at a central laboratory (ACM Global).

Efficacy parameters included UPCR determined in the first morning void samples at baseline and before each visit. Achievement of the FSGS partial remission endpoint (FPRE; UPCR ≤1.5 g/g and >40% decrease in UPCR^24^) was determined at each visit. eGFR was derived using the Modification of Diet in Renal Disease formula for patients aged ≥18 years^25^^,^^26^ and the revised Schwartz formula for patients aged <18 years.^27^ The proportions of patients reaching a confirmed 40% reduction in eGFR from the first sparsentan dose (consecutive eGFR results at least 28 days apart) or KF (study discontinuation included the term “end-stage kidney disease,” consecutive eGFR values <15 mL/min/1.73 m^2^ at least 14 days apart, or dialysis) were evaluated.

Safety was evaluated by the incidence of treatment-emergent AEs (TEAEs), treatment-related TEAEs, serious TEAEs, and TEAEs that led to study discontinuation. TEAEs were examined by yearly intervals and by the number of cases per 100 patient-years through the data cutoff. Physical examinations, vital signs (including BP), and safety laboratory parameters (eg, hemoglobin <9 g/dL, serum potassium concentrations 5.5-5.9 and >6.0 mmol/L, and alanine aminotransferase and aspartate aminotransferase liver function tests >3× the upper limit of normal) were measured at each visit. The proportion of patients requiring hospitalization and the most common reasons for hospitalizations were examined.

### Statistical Analyses

Data were analyzed from the first sparsentan dose administered in the double-blind period or open-label extension for participants initially assigned to irbesartan therapy through the February 5, 2021, data cutoff. Data are reported by weeks from first sparsentan dose, which combines the data from the related study visit for patients initially randomized to sparsentan and data from the next study visit for patients initially randomized to irbesartan because patients who received irbesartan did not begin sparsentan treatment until week 8 (eg, the 8 weeks from first sparsentan dose time point combines the week 8 visit data of patients initially randomized to sparsentan and the week 16 visit data of patients initially randomized to irbesartan so that both patient groups had an 8-week duration of sparsentan treatment). Statistical analyses were performed using SAS version 9.4 (SAS Institute, Cary, NC). Proteinuria was examined as mean percent change from baseline in UPCR by visit and as the proportion of patients who achieved FPRE by visit. Chronic eGFR slope in defined FPRE patient subgroups was determined via a mixed model with random coefficients (patient-specific slopes and intercepts) and linear spline (ie, a 2-slope model with knot or change point at week 6; Supplementary Methods). Kaplan-Meier analyses examined time to treatment discontinuation and time to confirmed 40% reduction in eGFR or KF. The acute effects of transition to sparsentan following RAASi washout (double-blind sparsentan group, from first dose in the double-blind period) versus no washout (double-blind irbesartan group, from first dose in the open-label extension) from baseline through week 16 in proteinuria, eGFR, and systolic and diastolic BP were examined descriptively (Supplementary Methods). Safety data are summarized descriptively.

## Results

### Patient Disposition

The DUET study enrolled 109 patients between April 2014 and April 2016, including 23 patients aged 8-18 years. A total of 108 patients received ≥1 sparsentan dose in the double-blind period and/or open-label extension and are included in this analysis (median sparsentan dose by dose cohort and overall in Table S1). The median time to sparsentan treatment discontinuation at data cutoff was 3.9 years (95% confidence interval [CI], 2.6-5.2). Of the 73 patients originally randomized to sparsentan, 68 continued sparsentan in the open-label extension. Of the 36 patients randomized to receive irbesartan, 35 transitioned to sparsentan in the open-label extension. At the data cutoff, 45 patients were continuing sparsentan treatment (Table 1).

**Table 1:** Patient Disposition by Yearly Intervals

	All Sparsentan (N = 108)				
	Year 0 to <1	Year 1 to <2	Year 2 to <3	Year 3 to <4	Year 4 to <5
Ongoing	85 (78.7%)	72 (66.7%)	60 (55.6%)	54 (50.0%)	47 (43.5%)^a^
Discontinued	23 (21.3%)	13 (12.0%)	12 (11.1%)	6 (5.6%)	7 (6.5%)
Adverse event	10 (9.3%)	2 (1.9%)	2 (1.9%)	3 (2.8%)	3 (2.8%)
Lost to follow-up	3 (2.8%)	1 (0.9%)	0 (0%)	0 (0%)	0 (0%)
Other	2 (1.9%)	2 (1.9%)	0 (0%)	0 (0%)	0 (0%)
Physician decision	2 (1.9%)	2 (1.9%)	5 (4.6%)	1 (0.9%)	0 (0%)
Pregnancy	2 (1.9%)	1 (0.9%)	0 (0%)	1 (0.9%)	0 (0%)
Protocol deviation	1 (0.9%)	0 (0%)	1 (0.9%)	0 (0%)	0 (0%)
Withdrawal by patient	3 (2.8%)	5 (4.6%)	3 (2.8%)	1 (0.9%)	2 (1.9%)
Noncompliance with study drug	0 (0%)	0 (0%)	1 (0.9%)	0 (0%)	1 (0.9%)
Missing	0 (0%)	0 (0%)	0 (0%)	0 (0%)	1 (0.9%)

*Note:* Data are given as n (%). Year 0 to <1 begins at DUET study baseline for patients initially randomized to sparsentan or at week 8 at the start of sparsentan treatment in the open-label extension for patients initially randomized to irbesartan during the double-blind period.

a Forty-five patients were ongoing at data cutoff at 4.6 years.

Slightly more than half of the patients were male sex (55.6%); most reported White (75.9%) or Black or African American (13.9%) race and were not Hispanic or Latino (82.4%; Table 2). Before the first sparsentan dose (ie, study baseline for patients initially randomized to sparsentan and before the open-label extension for patients initially randomized to irbesartan), median UPCR was 2.7 g/g, and mean eGFR was 74.5 mL/min/1.73 m^2^.

**Table 2:** Patient Characteristics

Characteristic	All Sparsentan (N = 108)	SPAR:SPAR (n = 73)	IRB:SPAR (n = 35)
Age (y)			
Mean ± SD	36.9 ± 16.5	38.0 ± 16.8	34.5 ± 16.0
Median (min, max)	39.0 (8, 71)	40.0 (8, 71)	35.0 (8, 67)
Age <18 y	18 (16.7%)	11 (15.1%)	7 (20.0%)
Sex			
Male	60 (55.6%)	41 (56.2%)	19 (54.3%)
Female	48 (44.4%)	32 (43.8%)	16 (45.7%)
Race			
Asian	6 (5.6%)	5 (6.8%)	1 (2.9%)
Black or African American	15 (13.9%)	8 (11.0%)	7 (20.0%)
White	82 (75.9%)	57 (78.1%)	25 (71.4%)
Other^a^	5 (4.6%)	3 (4.1%)	2 (5.7%)
Ethnicity			
Not Hispanic or Latino	89 (82.4%)	59 (80.8%)	30 (85.7%)
Hispanic or Latino	19 (17.6%)	14 (19.2%)	5 (14.3%)
Age at FSGS diagnosis (y)			
Mean ± SD	33 ± 16.4	34.4 ± 16.5	30.1 ± 16.1
Median (IQR)	33.3 (18.1, 46.7)	34.3 (21.3, 46.8)	30.2 (15.0, 46.6)
Time from FSGS diagnosis to informed consent (y)			
Mean ± SD	4.3 ± 4.5	4.1 ± 4.4	4.8 ± 4.8
Median (IQR)	2.5 (1.1, 6.0)	2.3 (1.1, 5.9)	3.4 (1.1, 6.9)
Before first sparsentan dose^b^			
Systolic BP (mm Hg)	129.0 ± 12.4	131.1 ± 11.4	124.4 ± 13.1
Diastolic BP (mm Hg)	81.6 ± 8.8	82.7 ± 8.9	79.4 ± 8.4
UPCR (g/g)			
Mean ± SD	3.5 ± 2.9	3.9 ± 3.3	2.8 ± 1.9
Median (min, max)^a^	2.7 (0.3, 14.0)	2.9 (0.3, 14.0)	2.3 (0.4, 10.1)
eGFR (mL/min/1.73 m^2^)			
Mean ± SD	74.5 ± 39.1	74.4 ± 37.3	74.7 ± 43.0
Median (min, max)^c^	69.4 (25.7, 215.9)	73.4 (27.6, 192.3)	63.1 (25.7, 215.9)
Hemoglobin (g/dL)	13.1 ± 2.0	13.3±1.9	12.7±2.1
Plasma lipid profile (mg/dL)			
Total cholesterol	261.1 ± 95.7	270.9 ± 106.7	240.5 ± 63.8
HDL cholesterol	55.8 ± 19.8	55.4 ± 21.6	56.8 ± 15.8
LDL cholesterol	159.8 ± 70.2	164.6 ± 76.5	149.9 ± 54.5
ALT (U/L)	90.0 ± 49.7	85.0 ± 36.3	100.5 ± 69.4
AST (U/L)	24.2 ± 11.2	23.9 ± 11.7	24.8 ± 10.2
At DUET study baseline			
Nephrotic range UPCR (adult, >3.5 g/g; pediatric, >2.0 g/g)	55 (50.9%)	40 (54.8%)	15 (42.9%)
Documented nephrotic syndrome in medical history or baseline	23 (21.3%)	16 (21.9%)	7 (20.0%)
Medications			
Any immunosuppressive treatment for kidney indications	35 (32.4%)	24 (32.9%)	11 (31.4%)
Steroids	17 (15.7%)	15 (20.5%)	2 (5.7%)
Calcineurin inhibitors	19 (17.6%)	14 (19.2%)	5 (14.3%)
Mycophenolate mofetil	13 (12.0%)	6 (8.2%)	7 (20.0%)
≥1 diuretic or antihypertensive agent	59 (54.6%)	40 (54.8%)	19 (54.3%)
Diuretic use	38 (35.2%)	30 (41.1%)	8 (22.9%)
Additional antihypertensive treatments (not RAASi)	40 (37.0%)	26 (35.6%)	14 (40.0%)

*Note:* Data are given as mean ± SD or n (%) unless otherwise noted.

Abbreviations: ALT, alanine aminotransferase; AST, aspartate aminotransferase; BP, blood pressure; eGFR, estimated glomerular filtration rate; FSGS, focal segmental glomerulosclerosis; HDL, high-density lipoprotein; IQR, interquartile range; IRB, irbesartan; LDL, low-density lipoprotein; RAASi, renin-angiotensin-aldosterone system inhibitor; SD, standard deviation; SPAR, sparsentan; UPCR, urinary protein-creatinine ratio.

a Other race includes patient responses of multiracial, Hispanic only, Egyptian, and unknown.

b DUET study baseline for patients initially randomized to sparsentan or at week 8 at the start of the open-label extension for patients initially randomized to irbesartan during the double-blind period. The SPAR:SPAR and IRB:SPAR groups should not be directly compared on values before the first sparsentan dose: the SPAR:SPAR group had a washout of RAASi before the first dose of sparsentan, whereas the IRB:SPAR group received maximized irbesartan and no RAASi washout before the first sparsentan dose in the open-label extension.

c eGFR was determined using the Chronic Kidney Disease Epidemiology formula for patients ≥18 years of age at screening and the Modified Schwartz formula for patients <18 years of age at screening.

Concomitant medications during sparsentan treatment were examined at yearly intervals (Table 3). Use of steroids and mycophenolate mofetil declined over time, whereas calcineurin inhibitor use was stable. Use of additional antihypertensive (non-RAASi) treatments and diuretics was stable. Use of medication to treat hyperkalemia was stable in the first 3 years of follow-up and then increased in the final 2 years.

**Table 3:** Concomitant Medications During Sparsentan Treatment by Yearly Intervals

	Number Within Each Year				
	Year 0 to <1 (n = 108)	Year 1 to <2 (n = 87)	Year 2 to <3 (n = 72)	Year 3 to <4 (n = 60)	Year 4 to <5 (n = 54)
Immunosuppressant treatment for kidney indications	39 (36.1%)	28 (32.2%)	21 (29.2%)	16 (26.7%)	13 (24.1%)
Steroids	21 (19.4%)	11 (12.6%)	6 (8.3%)	3 (5.0%)	3 (5.6%)
Calcineurin inhibitors	20 (18.5%)	16 (18.4%)	13 (18.1%)	12 (20.0%)	11 (20.4%)
Mycophenolate mofetil	15 (13.9%)	9 (10.3%)	8 (11.1%)	5 (8.3%)	4 (7.4%)
Lipid-lowering medications	54 (50.0%)	46 (52.9%)	43 (59.7%)	38 (63.3%)	35 (64.8%)
Additional antihypertensive treatments (including diuretics; not including RAASi)	67 (62.0%)	53 (60.9%)	45 (62.5%)	38 (63.3%)	34 (63.0%)
Calcium channel blockers	32 (29.6%)	26 (29.9%)	21 (29.2%)	17 (28.3%)	15 (27.8%)
Beta blocking agents	23 (21.3%)	18 (20.7%)	17 (23.6%)	14 (23.3%)	10 (18.5%)
Diuretic medications	50 (46.3%)	39 (44.8%)	33 (45.8%)	28 (46.7%)	24 (44.4%)
Sulfonamides	42 (38.9%)	32 (36.8%)	29 (40.3%)	24 (40.0%)	20 (37.0%)
Thiazides	9 (8.3%)	7 (8.0%)	6 (8.3%)	4 (6.7%)	4 (7.4%)
Aldosterone antagonists	0 (0%)	2 (2.3%)	1 (1.4%)	1 (1.7%)	1 (1.9%)
Other potassium-sparing agents	1 (0.9%)	0 (0%)	0 (0%)	0 (0%)	0 (0%)
Hyperkalemia medications	4 (3.7%)	4 (4.6%)	2 (2.8%)	5 (8.3%)	7 (13.0%)
Sodium polystyrene sulfonate	3 (2.8%)	3 (3.4%)	1 (1.4%)	3 (5.0%)	4 (7.4%)
Patiromer sorbitex calcium	0 (0%)	1 (1.1%)	1 (1.4%)	2 (3.3%)	1 (1.9%)
Sodium zirconium cyclosilicate	0 (0%)	0 (0%)	0 (0%)	1 (1.7%)	2 (3.7%)
Calcium polystyrene sulfonate	1 (0.9%)	0 (0%)	0 (0%)	0 (0%)	0 (0%)

*Note:* Data are given as n (%). Sodium-glucose cotransporter 2 inhibitors are allowed during the open-label extension at the discretion of the Investigator. One patient ongoing in the open-label extension has been receiving the sodium-glucose cotransporter 2 inhibitor dapagliflozin since October 2020.

Abbreviation: RAASi, renin-angiotensin-aldosterone system inhibitor.

### Long-term Efficacy

**Figure 1:**
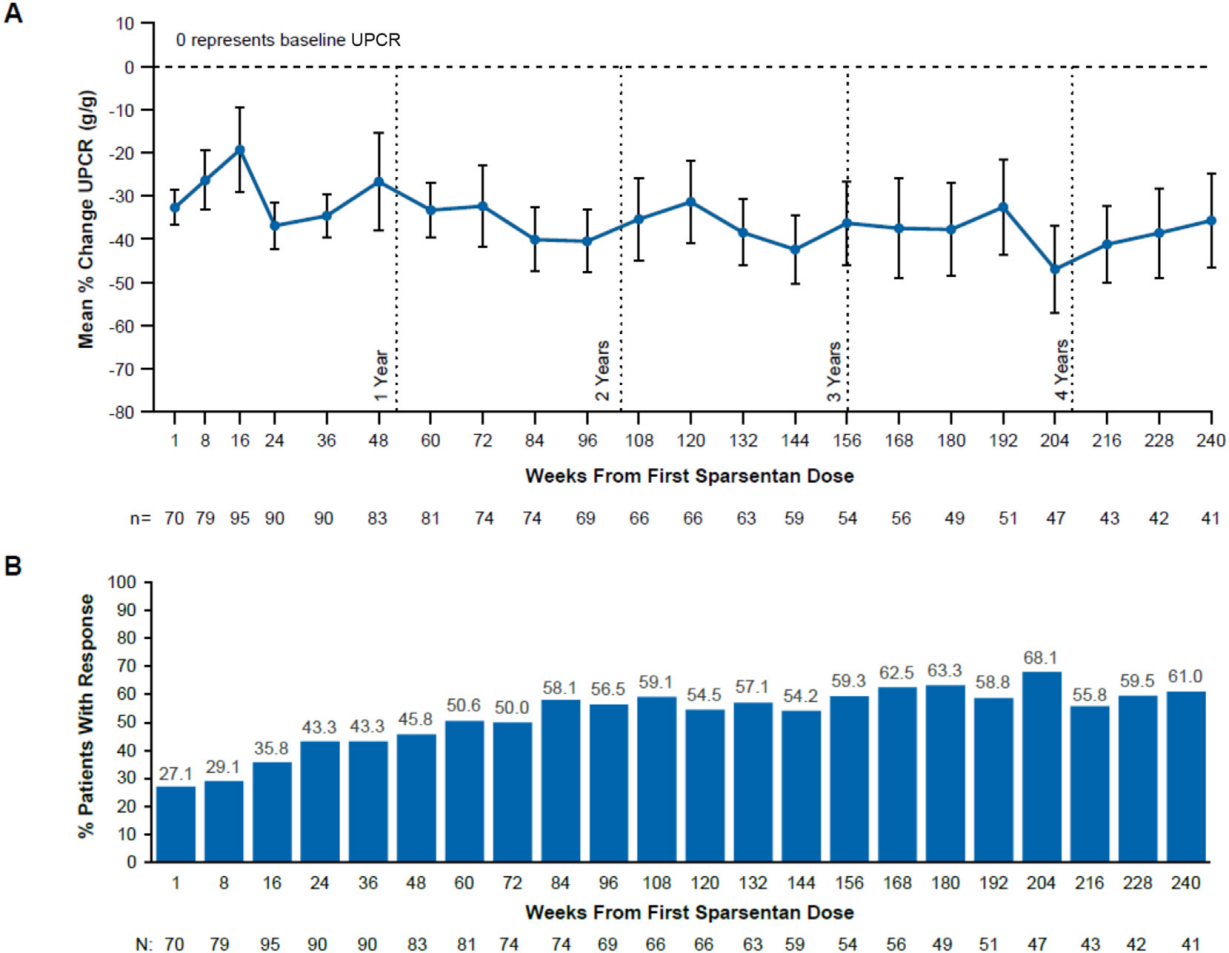
Change in urinary protein-creatinine ratio (UPCR) by weeks from first sparsentan dose as (A) mean percent change from baseline in UPCR and (B) patients achieving focal segmental glomerulosclerosis partial remission endpoint (FPRE). Error bars show standard error. Only on-treatment observations (defined as occurring within 1 day of last sparsentan dose) are included. FPRE is defined as UPCR ≤1.5 g/g and >40% reduction in UPCR from baseline. Percentage is calculated as the number of patients achieving FPRE divided by the number of patients with available FPRE results at the time point. ^a^Baseline for patients initially randomized to sparsentan is the DUET study baseline and for patients initially randomized to irbesartan is at week 8 at the start of sparsentan treatment in the open-label extension. Data are reported by weeks from first sparsentan dose, which combines the data from the related study visit for patients initially randomized to sparsentan and data from the next study visit for patients initially randomized to irbesartan because patients who received irbesartan did not begin sparsentan treatment until week 8 (eg, the 8 weeks from first sparsentan dose time point combines the week 8 visit data of patients initially randomized to sparsentan and the week 16 visit data of patients initially randomized to irbesartan so that both patient groups had an 8-week duration of sparsentan treatment). There was no assessment time point at 1 week from first sparsentan dose for patients initially randomized to irbesartan.

Initiation of sparsentan treatment resulted in a rapid reduction in UPCR within 8 weeks, followed by a sustained antiproteinuric effect in mean percentage change from baseline (Fig 1A). This rapid decline in proteinuria in response to sparsentan was independent of initial treatment randomization, ie, was observed both in patients after RAASi washout and in patients who transitioned to sparsentan without washout (Fig S1A). The percentage of patients who achieved FPRE increased through the first year of sparsentan treatment and remained at ≥50% of patients after the first year (Fig 1B).

**Figure 2:**
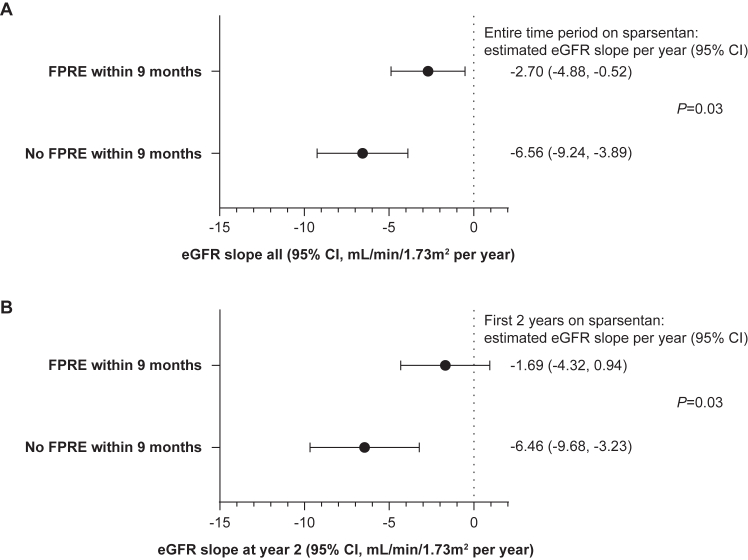
Chronic estimated glomerular filtration rate (eGFR) slope estimates (95% confidence interval [CI]) by focal segmental glomerulosclerosis partial remission endpoint (FPRE) group (achieved FPRE within 9 months of the first sparsentan dose versus did not achieve FPRE within 9 months) for (A) the entire time period on sparsentan treatment and (B) the first 2 years on sparsentan treatment.

**Figure 3:**
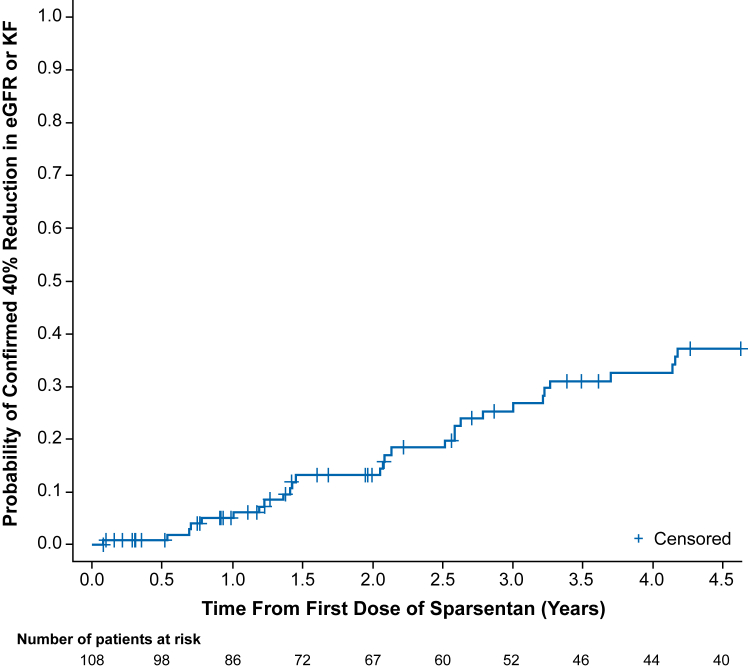
Time to confirmed 40% reduction in estimated glomerular filtration rate (eGFR) or kidney failure (KF). Baseline (time 0) for patients initially randomized to sparsentan is the DUET study baseline and for patients initially randomized to irbesartan is at week 8 at the start of sparsentan treatment in the open-label extension. Data are reported by time from first sparsentan dose, which combines the data from a study visit for patients initially randomized to sparsentan and data from the next study visit for patients initially randomized to irbesartan because patients who received irbesartan did not begin sparsentan treatment until week 8 (ie, so that both patient groups have the same duration of sparsentan treatment at each assessed time point). eGFR was determined using the Chronic Kidney Disease Epidemiology formula for patients ≥18 years of age at screening and the Modified Schwartz formula for patients <18 years of age at screening.

The patients initially randomized to sparsentan following RAASi washout showed similar acute transient reduction in eGFR versus patients who transitioned to sparsentan from irbesartan with no RAASi washout (Fig S1B). To test whether achieving FPRE within 9 months of the first sparsentan dose had an impact on kidney function, chronic eGFR slopes were compared between patients who achieved FPRE within 9 months and those who did not achieve FPRE within 9 months. Achieving FPRE within 9 months of first sparsentan dose (57/108; 52.8% of patients; Table S2) versus no FPRE within 9 months (51/108; 47.2%) was associated with a significantly slower annual rate of decline in eGFR over the entire treatment period (slope estimates, −2.70 vs −6.56 mL/min/1.73 m^2^ per year, respectively; *P* = 0.03; Fig 2A) and during the first 2 years (slope estimates, −1.69 vs −6.46 mL/min/1.73 m^2^ per year, respectively; *P* = 0.03; Fig 2B). Patient characteristics, sparsentan dose, and concomitant medications by FPRE within 9 months groups are shown in Tables S2-S4. A total of 33 patients (30.6%) reached the composite clinical endpoint of a confirmed 40% reduction in eGFR or KF over the follow-up period (Fig 3). The composite included 32 (29.6%) patients with a confirmed 40% reduction in eGFR and 12 (11.1%) patients who reached KF (all but 1 patient with KF also had a confirmed 40% reduction in eGFR). The Kaplan-Meier estimate for median time to first occurrence of the composite endpoint was not estimable at the data cutoff.

**Figure 4:**
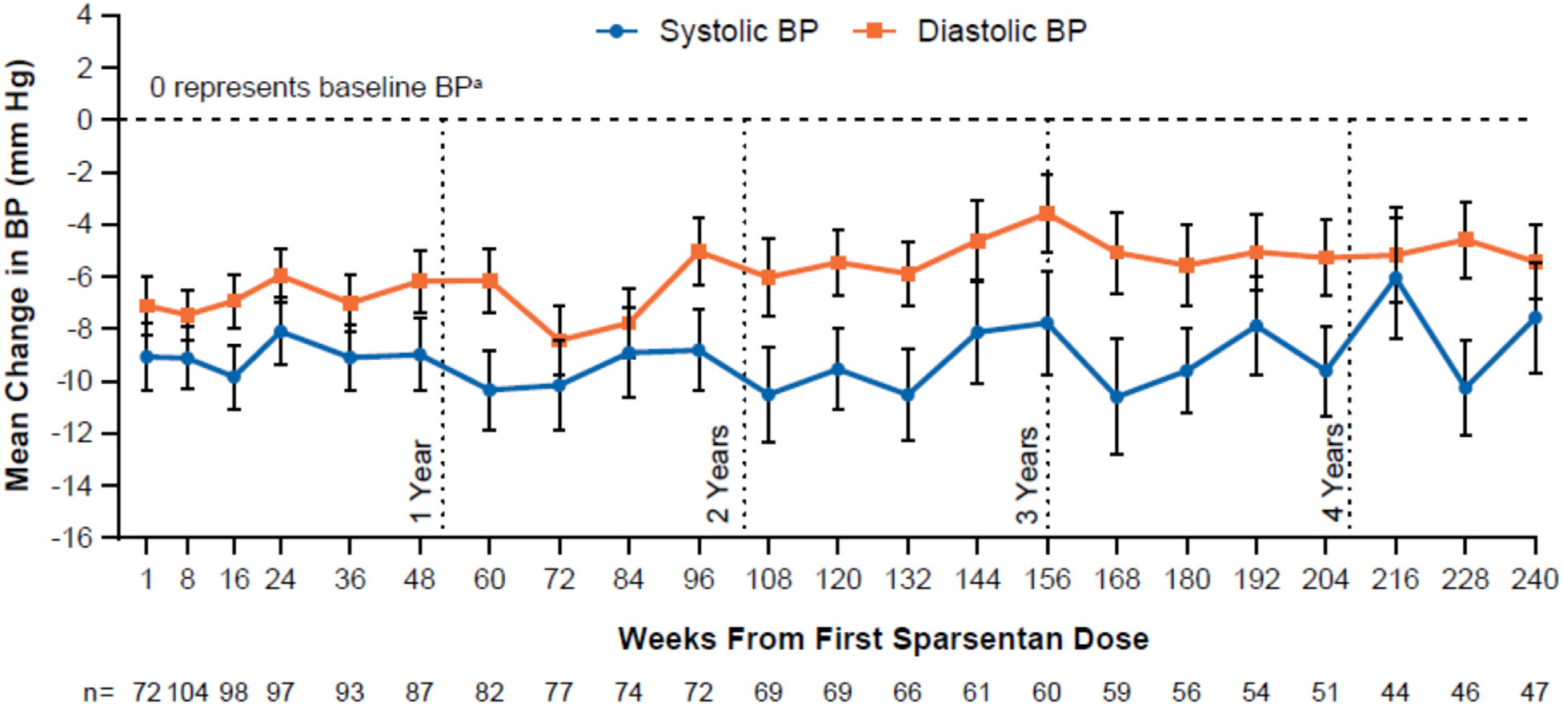
Mean change from baseline in systolic and diastolic blood pressure (BP) by weeks from first sparsentan dose. ^a^Baseline for patients initially randomized to sparsentan is the study baseline and for patients initially randomized to irbesartan is at week 8 at the start of sparsentan treatment in the open-label extension. Data are reported by weeks from first sparsentan dose, which combines the data from the related study visit for patients initially randomized to sparsentan and data from the next study visit for patients initially randomized to irbesartan because patients who received irbesartan did not begin sparsentan treatment until week 8 (eg, the 8 weeks from first sparsentan dose time point combines the week 8 visit data of patients initially randomized to sparsentan and the week 16 visit data of patients initially randomized to irbesartan so that both patient groups had an 8-week duration of sparsentan treatment). Error bars show standard error. Only on-treatment observations (defined as occurring within 1 day of last sparsentan dose) are included.

Sparsentan treatment led to a decrease in both systolic and diastolic BP within the first several weeks of treatment. BP then remained stable throughout the follow-up period (Fig 4). BP reduction was more prominent in patients initially randomized to sparsentan following RAASi washout than in the patients who transitioned to sparsentan without RAASi washout (Fig S1C).

### Long-term Safety

The most common TEAEs, with incidence >9 cases/100 patient-years, were headache, peripheral edema, upper respiratory infection, hyperkalemia, and hypotension (Table 4). A higher percentage of patients experienced TEAEs in the first year, with declines in subsequent yearly intervals for headache, peripheral edema, hypotension, dizziness, and anemia. In contrast, upper respiratory infection and hyperkalemia (Fig S2) were stable over the yearly intervals. TEAEs were similar in pediatric and adult patients (Table S5). The proportion of patients with hemoglobin <9 g/dL declined after the first year (5.9% at the week 8 visit) to 0% to ≤3.6% across visits. Alanine aminotransferase and/or aspartate aminotransferase >3× the upper limit of normal were reported in 4 patients (1 patient each at weeks 4 and 108; 2 patients at approximately week 36). Two of these patients discontinued treatment due to a liver-related TEAE, including 1 patient with a serious AE. The other 2 patients discontinued treatment before the data cutoff, 1 due to starting dialysis and 1 for not responding to treatment. There were no reports of heart failure.

**Table 4:** Most Common TEAEs by Year and Cases per 100 Patient-Years for the Total Study Duration

	Number Within Each Year					Total Study Duration Cases Per 100 Patient-Years
	Year 0 to <1 (n = 108)	Year 1 to <2 (n = 87)	Year 2 to <3 (n = 72)	Year 3 to <4 (n = 60)	Year 4 to <5 (n = 54)	
Headache	25 (23.1%)	5 (5.7%)	1 (1.4%)	4 (6.7%)	2 (3.7%)	11.7
Peripheral edema	15 (13.9%)	10 (11.5%)	3 (4.2%)	2 (3.3%)	2 (3.7%)	11.2
Upper respiratory tract infection	9 (8.3%)	5 (5.7%)	6 (8.3%)	5 (8.3%)	2 (3.7%)	10.6
Hyperkalemia	7 (6.5%)	9 (10.3%)	3 (4.2%)	6 (10.0%)	6 (11.1%)	10.4
Hypotension	17 (15.7%)	6 (6.9%)	3 (4.2%)	2 (3.3%)	1 (1.9%)	9.3
Nausea	17 (15.7%)	3 (3.4%)	2 (2.8%)	4 (6.7%)	1 (1.9%)	8.5
Hypertension	6 (5.6%)	7 (8.0%)	2 (2.8%)	3 (5.0%)	6 (11.1%)	7.6
Vomiting	12 (11.1%)	2 (2.3%)	5 (6.9%)	2 (3.3%)	1 (1.9%)	7.6
Diarrhea	14 (13.0%)	3 (3.4%)	3 (4.2%)	1 (1.7%)	4 (7.4%)	7.1
Dizziness	14 (13.0%)	3 (3.4%)	1 (1.4%)	2 (3.3%)	0 (0%)	6.3
Blood creatinine increased	11 (10.2%)	1 (1.1%)	4 (5.6%)	0 (0%)	1 (1.9%)	5.5
Blood creatine phosphokinase increased	8 (7.4%)	2 (2.3%)	0 (0%)	3 (5.0%)	2 (3.7%)	4.9
Anemia	11 (10.2%)	1 (1.1%)	0 (0%)	2 (3.3%)	1 (1.9%)	4.1

*Note:* Data are given as n (%).

Abbreviation: TEAE, treatment-emergent adverse event.

The most common treatment-related TEAEs (ie, >4 cases/100 patient-years) were hyperkalemia, hypotension, and dizziness (Table S6). Treatment-related hyperkalemia was stable across the yearly intervals, whereas hypotension and dizziness declined after the first year.

Serious TEAEs that occurred in ≥2 patients are shown in Table S7. During the follow-up, 27 patients (25.0%) had ≥1 hospitalization. TEAEs that led to hospitalization in ≥2 patients included acute kidney injury (n = 6), chest pain (n = 3), pneumonia (n = 3), atrial fibrillation (n = 2), fluid overload (n = 2), and hyperkalemia (n = 2). There were no patient deaths while patients were receiving sparsentan.

Over the total study duration, 20 patients (18.5%) discontinued due to TEAEs, most commonly during the first year (Table 1). TEAEs that led to discontinuation in ≥2 patients were decreased glomerular filtration rate (n = 5), increased blood creatinine (n = 3), pregnancy (n = 3), acute kidney injury (n = 2), and increased hepatic enzyme (n = 2).

## Discussion

In this post hoc analysis of the ongoing DUET open-label extension, long-term sparsentan treatment in patients with FSGS resulted in a sustained antiproteinuric effect in mean percentage change from baseline in parallel with good tolerability and a favorable safety profile. Patients who remained on sparsentan during the ongoing DUET open-label extension on average showed substantial, sustained, and clinically meaningful proteinuria reduction during long-term treatment. FPRE, which has been shown to predict favorable clinical outcomes in FSGS,^24^ was achieved by almost one-third of patients at 8 weeks of sparsentan treatment and by ≥50% of patients at each visit from approximately 1 year of sparsentan treatment through follow-up. Indeed, patients who achieved FPRE within 9 months of the first sparsentan dose had markedly slower declines in kidney function compared with patients who did not achieve FPRE within 9 months. An analysis of the DUET trial and open-label extension that focused on complete proteinuria remission demonstrated that complete remission at least once at any time was achieved by 43% of patients, and it also was associated with a slower rate of decline in eGFR.^28^

The antiproteinuric and potentially nephroprotective effects of sparsentan in FSGS likely result from dual antagonism of ET_A_R and AT_1_R.^16^, ^17^, ^18^ In animal models of FSGS, actions of sparsentan have included protection of podocyte number, structure, and function, reduced glomerulosclerosis, rapid and sustained reduction in proteinuria, and preservation of glomerular filtration rate.^29^, ^30^, ^31^ Sparsentan also acts on other glomerular cells, reducing inflammation and protecting the endothelial glycocalyx, leading to reduced sclerotic damage and preserved glomerular function in models of FSGS and other kidney diseases.^29^, ^30^, ^31^, ^32^, ^33^, ^34^, ^35^ Sparsentan treatment in patients with FSGS in DUET reduced BP, and this may have partly contributed to the antiproteinuric effect; however, BP reductions stabilized within a few weeks following sparsentan initiation, and further reductions in proteinuria over long-term treatment occurred without parallel changes in BP.

There is no control arm in open-label extensions, and it is challenging to interpret nephroprotective efficacy. One potential approach is to compare the rate of eGFR decline in DUET open-label extension patients to published studies. In a cohort of 281 adult patients with FSGS and a history of nephrotic syndrome from the Toronto Registry, the overall population rate of eGFR decline was −6.48 ± 9.96 mL/min/1.73 m^2^/y (median follow-up, 65 months).^36^ eGFR slope in patients with partial remission, characterized by traditional criteria (ie, >50% reduction in peak proteinuria and <3.5 g/d) was −5.64 ± 7.80 mL/min/1.73 m^2^/y. Data were collected during the 1980s-1990s, with presumably more limited therapeutic options. In the FSGS-CT cohort of 138 pediatric and young adult patients with steroid-resistant FSGS, eGFR decline (measured from week 26 of IST) was −6.7 mL/min/1.73 m^2^/y (95% CI, −10.0 to −3.4) in patients with no proteinuria reduction; −4.0 mL/min/1.73 m^2^/y (95% CI, −6.4 to −1.6) in patients with 50% proteinuria reduction; and an increase of +2.3 mL/min/1.73 m^2^/y (95% CI, −4.0 to +5.0) in patients with >90% proteinuria reduction.^37^ However, the 24-month eGFR slopes measured from randomization were markedly steeper in all proteinuria response categories. In a third study, the pooled analysis (n = 482; follow-up = 5 years) of patients with FSGS from the FSGS-CT, NEPTUNE consortium, and Kidney Research Network had eGFR slopes as follows: adults −1.71 mL/min/1.73 m^2^/y (95% CI, −3.23 to −0.19); adolescents −3.84 mL/min/1.73 m^2^/y (95% CI, −5.86 to −1.82); and children −3.32 mL/min/1.73 m^2^/y (95% CI, −5.13 to −1.51).^38^ Shallow eGFR slopes were at least in part attributable to the large proportion of Kidney Research Network patients with slow progression. In the current DUET study analysis, patients who achieved FPRE within the first 9 months of sparsentan treatment had an annual eGFR chronic slope over the entire treatment period of −2.70 mL/min/1.73 m^2^/y and an annual eGFR chronic slope over the first 2 years of treatment of −1.69 mL/min/1.73 m^2^/y. We cannot assert conclusively that the favorable outcome compared with that of previous reports was due to sparsentan, but the findings are encouraging.

The overall safety profile of patients receiving sparsentan in the ongoing DUET open-label extension supports long-term treatment. No unexpected safety outcomes emerged during the open-label extension. The observed TEAEs correspond to known effects of angiotensin receptor blockers and endothelin receptor antagonists. Treatment discontinuation and the incidence of TEAEs were highest during the first year of follow-up, and the lack of titration to the target dose of sparsentan may have been a contributing factor. Incidence of TEAEs declined substantially during the follow-up. This differentiates sparsentan from IST, which usually causes an accumulation of AEs over time. Among the TEAEs that led to treatment discontinuation, there were 2 due to hepatic enzyme elevations; both were asymptomatic, did not have bilirubin elevation, or meet Hy’s law criteria, and the patients had a complete recovery.

Among the most common treatment-related TEAEs during the DUET open-label extension were hyperkalemia, hypotension, and dizziness (considered hypotension-associated). The percentage of patients who experienced hyperkalemia was stable across yearly intervals. Serum potassium concentration level examined by visit supported stable clinical management of potassium levels and avoidance of hyperkalemia for most patients during the open-label extension. Hypotension-associated TEAEs were highest in the first year of treatment. This may be at least partly explained by the study design using a 2-week RAASi washout before the first dose of sparsentan in the double-blind period and an absence of sparsentan dose titration. Examination of the acute changes in BP from baseline to week 16 of sparsentan treatment, comparing patients who transitioned to sparsentan following RAASi washout (double-blind period sparsentan group) with patients who transitioned to sparsentan in the open-label extension from irbesartan with no washout (double-blind period irbesartan group) showed a smaller decline in BP in the latter group of patients. Initiation of sparsentan without RAASi washout will likely mitigate the occurrence of hypotension.

The low rate of peripheral edema TEAEs and no occurrences of heart failure during sparsentan treatment in the DUET open-label extension contrasts with the high rate of fluid retention, edema, and heart failure reported during treatment with endothelin receptor antagonists in patients with type 2 diabetes and proteinuria.^19^^,^^20^^,^^39^^,^^40^ Sparsentan is a single-molecule dual antagonist of endothelin and angiotensin receptors, and the higher affinity for AT_1_R relative to ET_A_R ensures an effective level of ET_A_R antagonism is always accompanied by complete AT_1_R antagonism.^17^^,^^41^ The sparsentan clinical data (ie, DUET; DUPLEX in FSGS; PROTECT in immunoglobulin A nephropathy) indicate effective proteinuria reduction without clinically significant fluid retention or edema.^22^^,^^42^^,^^43^

During the ongoing DUET open-label extension, extending to nearly 7 years for some patients, the number of patients who remained in the study and received sparsentan decreased. The DUET trial FSGS population was heterogeneous (eg, immunologically mediated disease vs genetic, degree of interstitial fibrosis).^22^ A limitation of the current post hoc analysis is that the patients who chose to remain in the study were likely those who responded well to sparsentan treatment. Additionally, by design, the open-label extension allows all patients to receive sparsentan and does not provide a long-term controlled comparison for changes in proteinuria and kidney function. Controlled comparison of 2-year antiproteinuric and nephroprotective efficacy with sparsentan was recently reported for FSGS and immunoglobulin A nephropathy.^44^^,^^45^

## Conclusions

Long-term treatment with sparsentan in patients with FSGS in the DUET open-label extension at 4.6 years showed sustained mean percentage reduction in proteinuria in patients who continued treatment with sparsentan. A consistent safety profile was observed with no new or unexpected AEs.

## References

[1] D’Agati V.D., Kaskel F.J., Falk R.J. (2011). Focal segmental glomerulosclerosis. N Engl J Med.

[2] De Vriese A.S., Wetzels J.F., Glassock R.J., Sethi S., Fervenza F.C. (2021). Therapeutic trials in adult FSGS: lessons learned and the road forward. Nat Rev Nephrol.

[3] Saran R., Li Y., Robinson B. (2016). US Renal Data System 2015 annual data report: epidemiology of kidney disease in the United States. Am J Kidney Dis.

[4] Spino C., Jahnke J.S., Selewski D.T., Massengill S., Troost J., Gipson D.S. (2016). Changing the paradigm for the treatment and development of new therapies for FSGS. Front Pediatr.

[5] Trachtman R., Sran S.S., Trachtman H. (2015). Recurrent focal segmental glomerulosclerosis after kidney transplantation. Pediatr Nephrol.

[6] Uffing A., Pérez-Sáez M.J., Mazzali M. (2020). Recurrence of FSGS after kidney transplantation in adults. Clin J Am Soc Nephrol.

[7] Wood E.L., Kwan L., Burrows J.E., Singh G., Veale J., Lum E.L. (2023). Early recurrence of focal segmental glomerulosclerosis in kidney transplant recipients: when to consider regifting. Transpl Rep.

[8] Reiser J., Nast C.C., Alachkar N. (2014). Permeability factors in focal and segmental glomerulosclerosis. Adv Chronic Kidney Dis.

[9] Rosenberg A.Z., Kopp J.B. (2017). Focal segmental glomerulosclerosis. Clin J Am Soc Nephrol.

[10] Sadowski C.E., Lovric S., Ashraf S. (2015). A single-gene cause in 29.5% of cases of steroid-resistant nephrotic syndrome. J Am Soc Nephrol.

[11] Hildebrandt F. (2015). Decade in review—genetics of kidney diseases: genetic dissection of kidney disorders. Nat Rev Nephrol.

[12] Kidney Disease: Improving Global Outcomes (KDIGO) Glomerular Diseases Work Group (2021). KDIGO 2021 clinical practice guideline for the management of glomerular diseases. Kidney Int.

[13] Sethna C.B., Gipson D.S. (2014). Treatment of FSGS in children. Adv Chronic Kidney Dis.

[14] Kohan D.E., Barton M. (2014). Endothelin and endothelin antagonists in chronic kidney disease. Kidney Int.

[15] Smeijer J.D., Kohan D.E., Webb D.J., Dhaun N., Heerspink H.J.L. (2021). Endothelin receptor antagonists for the treatment of diabetic and nondiabetic chronic kidney disease. Curr Opin Nephrol Hypertens.

[16] Komers R., Plotkin H. (2016). Dual inhibition of renin-angiotensin-aldosterone system and endothelin-1 in treatment of chronic kidney disease. Am J Physiol Regul Integr Comp Physiol.

[17] Trachtman H., Hogan J.J., Tesar V., Komers R. (2020). Sparsentan. dual angiotensin II AT1 receptor blocker and endothelin ETA receptor antagonist, treatment of focal segmental glomerulosclerosis, treatment of IgA nephropathy. Drugs Future.

[18] Benigni A., Buelli S., Kohan D.E. (2021). Endothelin-targeted new treatments for proteinuric and inflammatory glomerular diseases: focus on the added value to anti-renin-angiotensin system inhibition. Pediatr Nephrol.

[19] Heerspink H.J.L., Parving H.H., Andress D.L. (2019). Atrasentan and renal events in patients with type 2 diabetes and chronic kidney disease (SONAR): a double-blind, randomised, placebo-controlled trial. Lancet.

[20] de Zeeuw D., Coll B., Andress D. (2014). The endothelin antagonist atrasentan lowers residual albuminuria in patients with type 2 diabetic nephropathy. J Am Soc Nephrol.

[21] Dhaun N., MacIntyre I.M., Kerr D. (2011). Selective endothelin-A receptor antagonism reduces proteinuria, blood pressure, and arterial stiffness in chronic proteinuric kidney disease. Hypertension.

[22] Trachtman H., Nelson P., Adler S. (2018). DUET: a phase 2 study evaluating the efficacy and safety of sparsentan in patients with FSGS. J Am Soc Nephrol.

[23] Komers R., Gipson D.S., Nelson P. (2017). Efficacy and safety of sparsentan compared with irbesartan in patients with primary focal segmental glomerulosclerosis: randomized, controlled trial design (DUET). Kidney Int Rep.

[24] Troost J.P., Trachtman H., Nachman P.H. (2018). An outcomes-based definition of proteinuria remission in focal segmental glomerulosclerosis. Clin J Am Soc Nephrol.

[25] Levey A.S., Bosch J.P., Lewis J.B., Greene T., Rogers N., Roth D. (1999). A more accurate method to estimate glomerular filtration rate from serum creatinine: a new prediction equation. Modification of Diet in Renal Disease Study Group. Ann Intern Med.

[26] Levey A.S., Coresh J., Greene T. (2006). Using standardized serum creatinine values in the modification of diet in renal disease study equation for estimating glomerular filtration rate. Ann Intern Med.

[27] Schwartz G.J., Work D.F. (2009). Measurement and estimation of GFR in children and adolescents. Clin J Am Soc Nephrol.

[28] Trachtman H., Diva U., Murphy E., Wang K., Inrig J., Komers R. (2023). Implications of complete proteinuria remission at any time in focal segmental glomerulosclerosis: sparsentan DUET trial. Kidney Int Rep.

[29] Gyarmati G., Shroff U., Izuhara A., Komers R., Bedard P., Peti-Peterdi J. (2021). FC 016 sparsentan improves glomerular blood flow and augments protective tissue remodeling in mouse models of focal segmental glomerulosclerosis (FSGS). Nephrol Dial Transplant.

[30] Gyarmati G., Deepak S.K., Shroff U.N. (2022). Sparsentan improves glomerular endothelial and podocyte functions and augments protective tissue repair in a mouse model of focal segmental glomerulosclerosis (FSGS) [abstract]. J Am Soc Nephrol.

[31] Bedard P., Jenkinson C., Komers R. (2022). MO255: Sparsentan protects the glomerular basement membrane and glycocalyx, and attenuates proteinuria in a rat model of focal segmental glomerulosclerosis (FSGS) [abstract]. Nephrol Dial Transplant.

[32] Nagasawa H., Suzuki H., Jenkinson C. (2022). MO261: Sparsentan, the dual endothelin angiotensin receptor antagonist (DEARA), attenuates albuminuria and protects from the development of renal injury to a greater extent than losartan in the GDDY mouse model of IgA nephropathy: a 16-week study [abstract]. Nephrol Dial Transplant.

[33] Reily C., Moldoveanu Z., Pramparo T. (2021). The dual endothelin angiotensin receptor antagonist (DEARA) sparsentan protects from glomerular hypercellularity and associated immune/inflammatory gene network activity in a model of IgA nephropathy. J Am Soc Nephrol.

[34] Jenkinson C., Moldoveanu Z., Komers R. (2019). SAT-010 protective effects of sparsentan from proliferative glomerular injury induced by administration of human immune complexes in a murine model of experimental IgA nephropathy. Kidney Int Rep.

[35] Cosgrove D., Gratton M.A., Madison J. (2023). Dual inhibition of the endothelin and angiotensin receptor ameliorates renal and inner ear pathologies in Alport mice. J Pathol.

[36] Troyanov S., Wall C.A., Miller J.A., Scholey J.W., Cattran D.C. (2005). Toronto Glomerulonephritis Registry Group. Focal and segmental glomerulosclerosis: definition and relevance of a partial remission. J Am Soc Nephrol.

[37] Troost J.P., Trachtman H., Spino C. (2021). Proteinuria reduction and kidney survival in focal segmental glomerulosclerosis. Am J Kidney Dis.

[38] Gipson D.S., Troost J.P., Spino C. (2022). Comparing kidney health outcomes in children, adolescents, and adults with focal segmental glomerulosclerosis. JAMA Netw Open.

[39] Mann J.F., Green D., Jamerson K. (2010). Avosentan for overt diabetic nephropathy. J Am Soc Nephrol.

[40] Kohan D.E., Pritchett Y., Molitch M. (2011). Addition of atrasentan to renin-angiotensin system blockade reduces albuminuria in diabetic nephropathy. J Am Soc Nephrol.

[41] Murugesan N., Gu Z., Fadnis L. (2005). Dual angiotensin II and endothelin A receptor antagonists: synthesis of 2'-substituted N-3-isoxazolyl biphenylsulfonamides with improved potency and pharmacokinetics. J Med Chem.

[42] Heerspink H.J.L., Radhakrishnan J., Alpers C.E. (2023). Sparsentan in patients with IgA nephropathy: a prespecified interim analysis from a randomised, double-blind, active-controlled clinical trial. Lancet.

[43] (Published May 1, 2023). Travere Therapeutics announces topline results from two-year primary efficacy endpoint in pivotal phase 3 DUPLEX study of sparsentan in focal segmental glomerulosclerosis.

[44] Rheault M.N., Alpers C.E., Barratt J. (2023). Sparsentan versus irbesartan in focal segmental glomerulosclerosis. N Engl J Med.

[45] Rovin B.H., Barratt J., Heerspink H.J.L. (2023). Efficacy and safety of sparsentan versus irbesartan in patients with IgA nephropathy (PROTECT): 2-year results from a randomised, active-controlled, phase 3 trial. Lancet.

