# Supplementary material for: Sparsentan for Focal Segmental Glomerulosclerosis in the DUET Open-Label Extension: Long-term Efficacy and Safety
Source: Kidney Med. 2024 Apr 26;6(6):100833. doi: 10.1016/j.xkme.2024.100833 (PMC11145552; doi:10.1016/j.xkme.2024.100833)
Supplement: Supplementary File (PDF) — Figures S1 and S2; Tables S1–S7. [file mmc1.pdf]

SUPPLEMENTARY MATERIAL

Supplementary Methods

Chronic estimated glomerular filtration rate (eGFR) slope in defined focal segmental glomerulosclerosis (FSGS) partial remission endpoint (FPRE) patient subgroups was determined via a mixed model with random coefficients (patient-specific slopes and intercepts) and linear spline (ie, a 2-slope model with knot or change point at week 6). Only eGFR values obtained while the patient was on sparsentan were included (ie, for patients receiving irbesartan in the double-blind period of the study, only eGFR values from the open-label extension period were included). Fixed effects in the model included baseline eGFR (relative to first dose of sparsentan), time (in days) from first dose of sparsentan, and time (in days) from change point at 42 days or 0, whichever was greater, and for FPRE within 9 months or no FPRE within 9 months group (and difference), numeric indicator of response and interaction terms for time from first dose of sparsentan by indicator and time from change point by indicator. Random effects for intercept, time from first dose of sparsentan, and time from change point were also included in the model. Unstructured covariance matrix was assumed.

The acute effects of transition to sparsentan following renin-angiotensin-aldosterone system inhibitors (RAASi) washout (DUET trial double-blind sparsentan group, from first dose in the double-blind period) versus no RAASi washout (DUET trial double-blind irbesartan group, from first sparsentan dose in the open-label extension) were examined from baseline at first sparsentan dose through the week 16 visit for acute changes in proteinuria, eGFR, and systolic and diastolic blood pressure (BP). Rapid decline in proteinuria in response to sparsentan was independent of initial randomization, ie, was observed both in patients after RAASi washout and in patients treated with irbesartan during the double-blind period who transitioned to sparsentan without RAASi washout (**Fig S1A**). The patients who were randomized to sparsentan following RAASi washout demonstrated comparable acute transient reduction in eGFR versus patients who transitioned to sparsentan from double-blind irbesartan treatment with no RAASi washout (**Fig S1B**). The acute reduction in BP was more prominent in patients randomized to sparsentan following RAASi washout versus the patients who transitioned to sparsentan from maximized RAASi with irbesartan and without RAASi washout (**Fig S1C**). This suggests that the initiation of sparsentan without RAASi washout will likely mitigate the occurrence of hypotension.

Figure S1. Acute changes with and without RAASi washout prior to first sparsentan dose by double-blind randomized treatment group in (A) acute mean percent change from baseline in UP/C; (B) acute mean change from baseline in eGFR, and (C) acute mean change from baseline in systolic and diastolic BP.

A

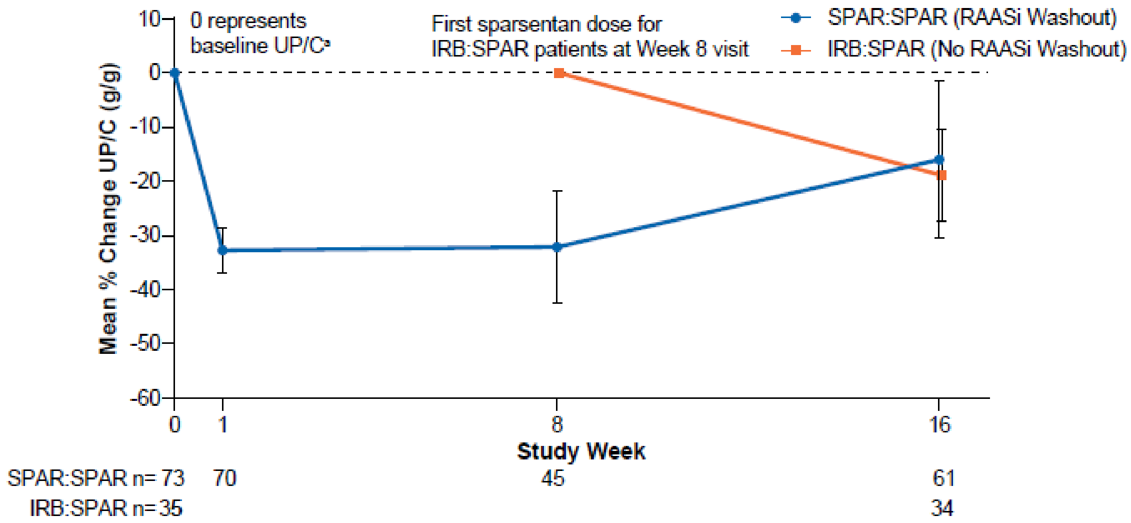

B

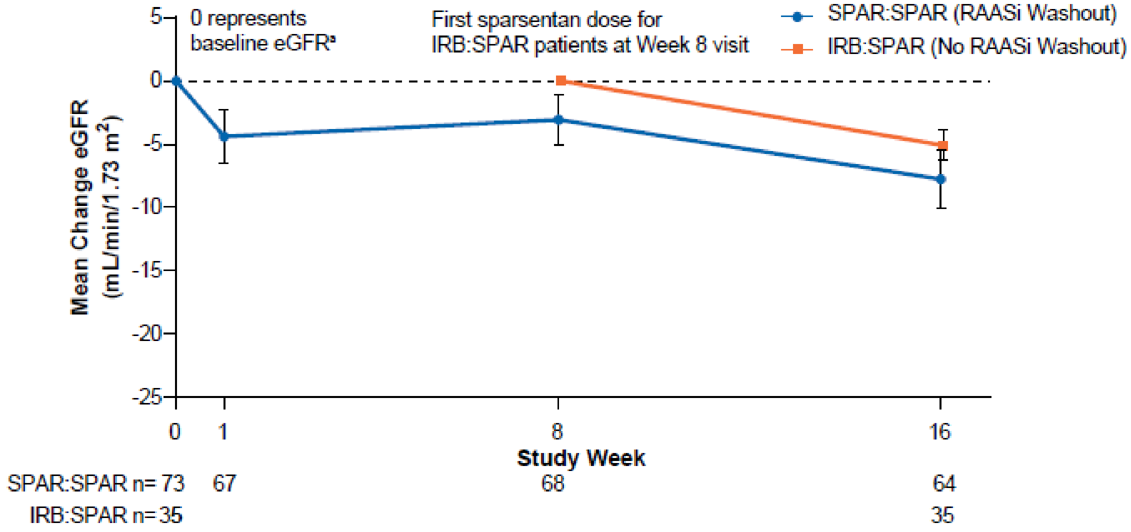

C1

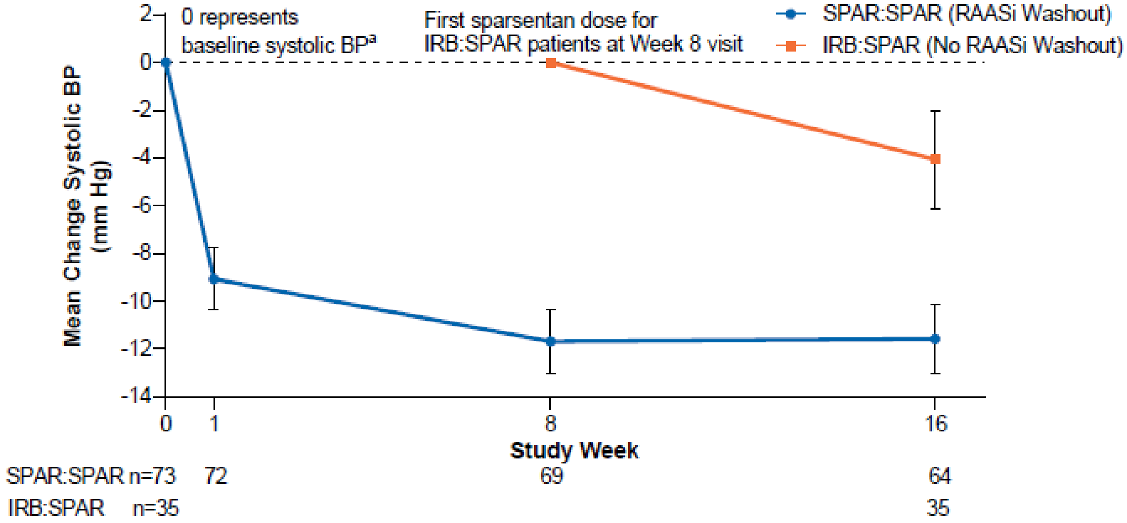

C2

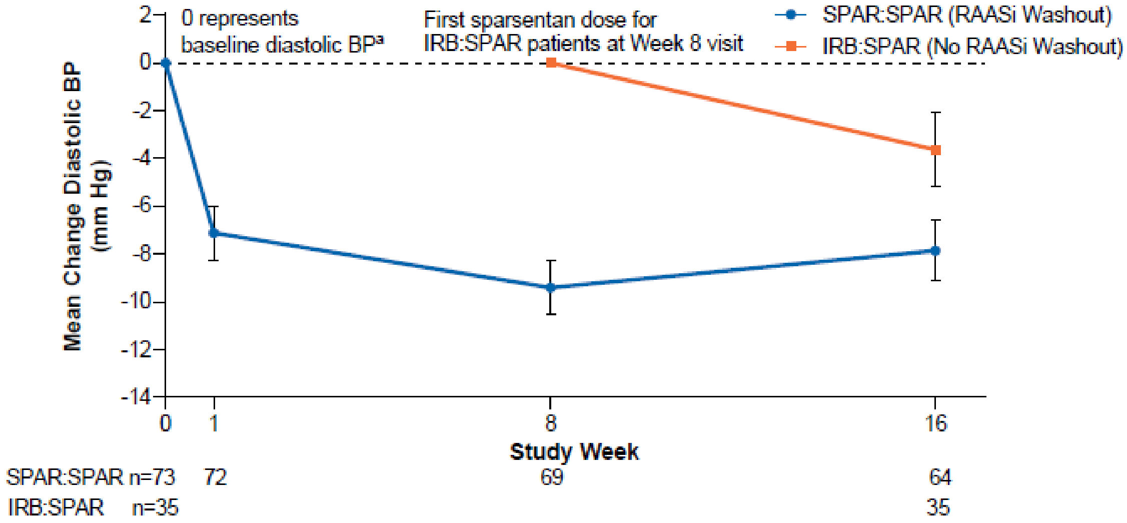

Error bars show standard error. Only on-treatment observations (defined as occurring within 1 day of last sparsentan dose) are included. eGFR was determined using the Chronic Kidney Disease Epidemiology formula for patients ≥18 years of age at screening, and the Modified Schwartz formula for patients <18 years of age at screening.

[a]Baseline for patients initially randomized to sparsentan in the DUET double-blind period is the study baseline and for patients initially randomized to irbesartan during the double-blind period is at week 8 at the start of sparsentan treatment in the open-label extension.

BP, blood pressure; eGFR, estimated glomerular filtration rate in mL/min/1.73m^2; IRB, irbesartan; RAASi, renin-angiotensin-aldosterone system inhibitors; SPAR, sparsentan; UP/C, urine protein/creatinine ratio.

Figure S2. Serum potassium concentration level by weeks from first sparsentan dose.

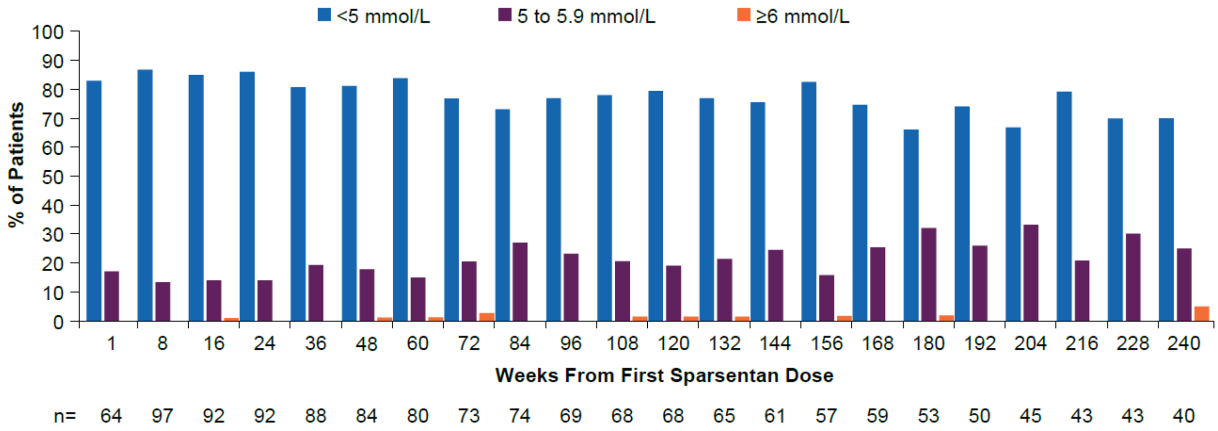

Data are reported by weeks from first sparsentan dose which combines the data from the related study visit for patients initially randomized to sparsentan and data from the next study visit for patients initially randomized to irbesartan since patients who received irbesartan did not begin sparsentan treatment until week 8 (eg, the 8 weeks from first sparsentan dose timepoint combines the week 8 visit data of patients initially randomized to sparsentan and the week 16 visit data of patients initially randomized to irbesartan so that both patient groups had an 8-week duration of sparsentan treatment). There was no assessment timepoint at 1 week from first sparsentan dose for patients initially randomized to irbesartan.

Table S1. Median Sparsentan Dose by DUET Dose Cohort and Overall for the Total Study Duration

	**Sparsentan 200 mg/day (n=21)**	**Sparsentan 400 mg/day (n=45)**	**Sparsentan 800 mg/day (n=42)**	**All Sparsentan (N=108)**
**Median**	199.9	399.8	728.9	399.8
**Q1, Q3**	173.0, 205.0	336.0, 400.0	400.0, 799.6	229.1, 547.4
**Min, Max**	100.0, 582.3	198.3, 728.1	152.7, 800.0	100.0, 800.0

Table S2. Patient Characteristics by FPRE Within 9 Months of First Sparsentan Dose Groups

**Characteristic**	**FPRE Yes (n=57)**	**FPRE No (n=51)**
**Age, years**		
Mean±SD	39.5±17.1	33.9±15.4
Median (min, max)	41.0 (8, 71)	35.0 (8, 70)
**Age <18 years**	8 (14.0)	10 (19.6)
**Sex**		
Male	31 (54.4)	29 (56.9)
Female	26 (45.6)	22 (43.1)
**Race**		
Asian	2 (3.5)	4 (7.8)
Black or African American	9 (15.8)	6 (11.8)
White	43 (75.4)	39 (76.5)
Other[a]	3 (5.3)	2 (3.9)
**Ethnicity**		
Not Hispanic or Latino	48 (84.2)	41 (80.4)
Hispanic or Latino	9 (15.8)	10 (19.6)
**Age at FSGS diagnosis, years**		
Mean±SD	35.2±17.3	30.6±15.2
Median (IQR)	33.7 (22.9, 48.6)	30.2 (17.9, 41.8)
**Time from FSGS diagnosis to informed consent, years**		
Mean±SD	4.8±5.0	3.8±3.9
Median (IQR)	2.9 (1.1, 6.0)	2.3 (1.0, 5.4)
**Prior to first sparsentan dose[b]**		
Systolic BP, mmHg	129.7±12.7	128.2±12.0
Diastolic BP, mmHg	80.8±8.8	82.5±8.9
UP/C, g/g		
Mean±SD	2.4±1.8	4.8±3.5
Median (min, max)	1.9 (0.3, 11.1)	3.9 (0.4, 14.0)
**At DUET study baseline**		
eGFR, mL/min/1.73m^2		
Mean±SD	75.7±38.5	73.1±41.8
Median (min, max)[c]	69.8 (28, 189)	64.1 (28, 212)
Nephrotic range UP/C (>3.5 g/g)	16 (28.1)	36 (70.6)
Documented nephrotic syndrome in medical history or baseline	10 (17.5)	13 (25.5)
Medications		
Any IST for kidney indications	21 (36.8)	14 (27.5)
Steroids	7 (12.3)	10 (19.6)
Calcineurin inhibitors	12 (21.1)	7 (13.7)

Mycophenolate mofetil	9 (15.8)	4 (7.8)
≥1 diuretic or antihypertensive agent	27 (47.4)	32 (62.7)
Diuretic use	18 (31.6)	20 (39.2)
Additional antihypertensive treatments (not RAASi)	19 (33.3)	21 (41.2)

Note: Data are given as mean±SD or n (%) unless otherwise noted. FPRE is defined as UP/C ≤1.5 g/g and >40% reduction in UP/C from baseline. The FPRE Yes group achieved FPRE within 9 months of the first sparsentan dose, and the FPRE No group did not achieve FPRE within 9 months of first sparsentan dose.
[a]Other race includes patient responses of multiracial, Hispanic only, Egyptian, and unknown.
[b]DUET study baseline for patients initially randomized to sparsentan or at week 8 at the start of the open-label extension for patients initially randomized to irbesartan during the double-blind period.
[c]eGFR was determined using the Chronic Kidney Disease Epidemiology formula for patients ≥18 years of age at screening, and the Modified Schwartz formula for patients <18 years of age at screening.
Abbreviations: BP, blood pressure; eGFR, estimated glomerular filtration rate; FPRE, FSGS partial remission endpoint; FSGS, focal segmental glomerulosclerosis; IST, immunosuppressive treatment; RAASi, renin-angiotensin-aldosterone system inhibitors; SD, standard deviation; UP/C, urine protein/creatinine ratio.

Table S3. Median Sparsentan Dose by DUET Dose Cohort and Overall for the Total Study Duration by FPRE Within 9 Months Group

	**Sparsentan 200 mg/day**		**Sparsentan 400 mg/day**		**Sparsentan 800 mg/day**		**All Sparsentan**	
	**FPRE Yes (n=5)**	**FPRE No (n=16)**	**FPRE Yes (n=30)**	**FPRE No (n=15)**	**FPRE Yes (n=22)**	**FPRE No (n=20)**	**FPRE Yes (n=57)**	**FPRE No (n=51)**
**Median**	199.9	198.9	399.8	399.8	728.9	722.6	399.8	399.8
**Q1, Q3**	100.1, 200.0	173.1, 220.1	252.8, 400.0	399.8, 400.0	400.0, 799.6	400.0, 799.6	271.7, 533.5	200.0, 582.3
**Min, Max**	100.0, 355.6	109.0, 582.3	198.3, 728.1	200.0, 479.3	161.3, 800.0	152.7, 800.0	100.0, 800.0	109.0, 800.0

FPRE is defined as UP/C ≤1.5 g/g and >40% reduction in UP/C from baseline. The FPRE Yes group achieved FPRE within 9 months of the first sparsentan dose, and the FPRE No group did not achieve FPRE within 9 months of first sparsentan dose. FPRE, FSGS partial remission endpoint; FSGS, focal segmental glomerulosclerosis.

Table S4. Concomitant Medications During Sparsentan Treatment by Yearly Intervals and FPRE Within 9 Months Group

	Number Within Each Year									
	Year 0 to <1		Year 1 to <2		Year 2 to <3		Year 3 to <4		Year 4 to <5	
	FPRE Yes (n=57)	FPRE No (n=51)	FPRE Yes (n=53)	FPRE No (n=34)	FPRE Yes (n=45)	FPRE No (n=27)	FPRE Yes (n=37)	FPRE No (n=23)	FPRE Yes (n=34)	FPRE No (n=20)
**IST for kidney indications**	23 (40.4)	16 (31.4)	19 (35.8)	9 (26.5)	16 (35.6)	5 (18.5)	12 (32.4)	4 (17.4)_	10 (29.4)	3 (15.0)
Steroids	11 (19.3)	10 (19.6)	8 (15.1)	3 (8.8)	5 (11.1)	1 (3.7)	2 (5.4)	1 (4.3)	2 (5.9)	1 (5.0)
Calcineurin inhibitors	12 (21.1)	8 (15.7)	10 (18.9)	6 (17.6)	9 (20.0)	4 (14.8)	9 (24.3)	3 (13.0)	9 (26.5)	2 (10.0)
Mycophenolate mofetil	9 (15.8)	6 (11.8)	7 (13.2)	2 (5.9)	6 (13.3)	1 (3.7)	4 (10.8)	1 (4.3)	3 (8.8)	1 (5.0)
**Lipid-lowering medications**	28 (49.1)	26 (51.0)	25 (47.2)	21 (61.8)	26 (57.8)	17 (63.0)	23 (62.2)	15 (65.2)	20 (58.8)	15 (75.0)
**Additional antihypertensive treatments (including diuretics; not including RAASi)**	33 (57.9)	34 (66.7)	32 (60.4)	21 (61.8)	26 (57.8)	19 (70.4)	21 (56.8)	17 (73.9)	19 (55.9)	15 (75.0)
Calcium channel blockers	15 (26.3)	17 (33.3)	14 (26.4)	12 (35.3)	13 (28.9)	8 (29.6)	9 (24.3)	8 (34.8)	7 (20.6)	8 (40.0)
Beta blocking agents	11 (19.3)	12 (23.5)	9 (17.0)	9 (26.5)	9 (20.0)	8 (29.6)	8 (21.6)	6 (26.1)	5 (14.7)	5 (25.0)
**Diuretic medications**	25 (43.9)	25 (49.0)	25 (47.2)	14 (41.2)	20 (44.4)	13 (48.1)	14 (37.8)	14 (60.9)	13 (38.2)	11 (55.0)
Sulfonamides	22 (38.6)	20 (39.2)	23 (43.4)	9 (26.5)	18 (40.0)	11 (40.7)	12 (32.4)	12 (52.2)	11 (32.4)	9 (45.0)
Thiazides	3 (5.3)	6 (11.8)	2 (3.8)	5 (14.7)	3 (6.7)	3 (11.1)	2 (5.4)	2 (8.7)	2 (5.9)	2 (10.0)
Aldosterone antagonists	0 (0)	0 (0)	1 (1.9)	1 (2.9)	0 (0)	1 (3.7)	0 (0)	1 (4.3)	0 (0)	1 (5.0)
Other potassium-sparing agents	0 (0)	1 (2.0)	0 (0)	0 (0)	0 (0)	0 (0)	0 (0)	0 (0)	0 (0)	0 (0)
**Hyperkalemia medications**	0 (0)	4 (7.8)	2 (3.8)	2 (5.9)	1 (2.2)	1 (3.7)	3 (8.1)	2 (8.7)	4 (11.8)	3 (15.0)
Sodium polystyrene sulfonate	0 (0)	3 (5.9)	1 (1.9)	2 (5.9)	0 (0)	1 (3.7)	2 (5.4)	1 (4.3)	2 (5.9)	2 (10.0)
Patiromer sorbitex calcium	0 (0)	0 (0)	1 (1.9)	0 (0)	1 (2.2)	0 (0)	1 (2.7)	1 (4.3)	1 (2.9)	0 (0)
Sodium zirconium cyclosilicate	0 (0)	0 (0)	0 (0)	0 (0)	0 (0)	0 (0)	0 (0)	1 (4.3)	1 (2.9)	1 (5.0)

Calcium polystyrene sulfonate	0 (0)	1 (2.0)	0 (0)	0 (0)	0 (0)	0 (0)	0 (0)	0 (0)	0 (0)	0 (0)

Note: Data are given as n (%). FPRE is defined as UP/C ≤1.5 g/g and >40% reduction in UP/C from baseline. FPRE Yes group achieved FPRE within 9 months of the first sparsentan dose and FPRE No group did not achieve FPRE within 9 months of first sparsentan dose. FPRE, FSGS partial remission endpoint; FSGS, focal segmental glomerulosclerosis; IST, immunosuppressive treatment; RAASi, renin-angiotensin-aldosterone system inhibitors; UP/C, urine protein/creatinine ratio.

Table S5. Most Common TEAEs by Year and by Pediatric and Adult Age Groups

	Number Within Each Year									
	Year 0 to <1		Year 1 to <2		Year 2 to <3		Year 3 to <4		Year 4 to <5	
	Pediatric (n=18)	Adult (n=90)	Pediatric (n=15)	Adult (n=72)	Pediatric (n=12)	Adult (n=60)	Pediatric (n=9)	Adult (n=51)	Pediatric (n=7)	Adult (n=47)
Headache	7 (38.9)	18 (20.0)	1 (6.7)	4 (5.6)	0 (0)	1 (1.7)	0 (0)	4 (7.8)	0 (0)	2 (4.3)
Edema[a]	2 (11.1)	15 (16.7)	0 (0)	10 (13.9)	0 (0)	3 (5.0)	0 (0)	2 (3.9)	0 (0)	2 (4.3)
Upper respiratory tract infection	3 (16.7)	6 (6.7)	2 (13.3)	3 (4.2)	2 (16.7)	4 (6.7)	1 (11.1)	4 (7.8)	0 (0)	2 (4.3)
Hyperkalemia	1 (5.6)	6 (6.7)	2 (13.3)	7 (9.7)	1 (8.3)	2 (3.3)	1 (11.1)	5 (9.8)	0 (0)	6 (12.8)
Hypotension	1 (5.6)	16 (17.8)	0 (0)	6 (8.3)	0 (0)	3 (5.0)	1 (11.1)	1 (2.0)	0 (0)	1 (2.1)
Nausea	4 (22.2)	13 (14.4)	0 (0)	3 (4.2)	1 (8.3)	2 (3.3)	1 (11.1)	3 (5.9)	1 (14.3)	0 (0)
Hypertension	2 (11.1)	4 (4.4)	0 (0)	7 (9.7)	1 (8.3)	1 (1.7)	0 (0)	3 (5.9)	1 (14.3)	5 (10.6)
Vomiting	4 (22.2)	8 (8.9)	0 (0)	2 (2.8)	1 (8.3)	4 (6.7)	1 (11.1)	1 (2.0)	1 (14.3)	0 (0)
Diarrhea	2 (11.1)	12 (13.3)	0 (0)	3 (4.2)	1 (8.3)	2 (3.3)	0 (0)	1 (2.0)	1 (14.3)	3 (6.4)
Dizziness	2 (11.1)	12 (13.3)	0 (0)	3 (4.2)	0 (0)	1 (1.7)	1 (11.1)	1 (2.0)	0 (0)	0 (0)
Blood creatinine increased	2 (11.1)	9 (10.0)	0 (0)	1 (1.4)	2 (16.7)	2 (3.3)	0 (0)	0 (0)	0 (0)	1 (2.1)
Blood creatine phosphokinase increased	1 (5.6)	7 (7.8)	1 (6.7)	1 (1.4)	0 (0)	0 (0)	1 (11.1)	2 (3.9)	0 (0)	2 (4.3)
Anemia	3 (16.7)	8 (8.9)	0 (0)	1 (1.4)	0 (0)	0 (0)	1 (11.1)	1 (2.0)	0 (0)	1 (2.1)

[a]Pediatric (face); Adult (peripheral). *Note:* Data are given as n (%). Pediatric patients age <18 years old. Adult patients age ≥18 years old. Abbreviation: TEAEs, treatment-emergent adverse events.

Table S6. Most Common Treatment-Related TEAEs by Year and Cases per 100 Patient-Years for the Total Study Duration

	Number Within Each Year					
	**Year 0 to <1 (n=108)**	**Year 1 to <2 (n=87)**	**Year 2 to <3 (n=72)**	**Year 3 to <4 (n=60)**	**Year 4 to <5 (n=54)**	**Total Study Duration Cases Per 100 Patient-Years, Cases/100 Patient Years**
Hyperkalemia	6 (5.6)	8 (9.2)	3 (4.2)	5 (8.3)	5 (9.3)	9.3
Hypotension	14 (13.0)	5 (5.7)	2 (2.8)	2 (3.3)	1 (1.9)	7.9
Dizziness	10 (9.3)	3 (3.4)	0 (0)	1 (1.7)	0 (0)	4.1
Headache	11 (10.2)	1 (1.1)	0 (0)	0 (0)	0 (0)	3.8
Nausea	8 (7.4)	0 (0)	1 (1.4)	1 (1.7)	0 (0)	3.5
Blood creatinine increased	6 (5.6)	0 (0)	3 (4.2)	0 (0)	1 (1.9)	3.0
Edema peripheral	5 (4.6)	2 (2.3)	1 (1.4)	0 (0)	0 (0)	2.5
Glomerular filtration rate decreased	3 (2.8)	2 (2.3)	0 (0)	1 (1.7)	2 (3.7)	2.5
Vomiting	6 (5.6)	0 (0)	0 (0)	1 (1.7)	0 (0)	2.5
Anemia	6 (5.6)	0 (0)	0 (0)	1 (1.7)	0 (0)	1.9
Blood creatine phosphokinase increased	3 (2.8)	0 (0)	0 (0)	1 (1.7)	2 (3.7)	1.9
Acute kidney injury	2 (1.9)	1 (1.1)	1 (1.4)	0 (0)	1 (1.9)	1.6
Orthostatic hypotension	4 (3.7)	0 (0)	0 (0)	0 (0)	0 (0)	1.1

Note: Data are given as n (%).
Abbreviations: TEAE, treatment-emergent adverse event.

Table S7. Serious TEAEs in ≥2 Patients by Year and Cases per 100 Patient-Years for the Total Study Duration

	Number Within Each Year					
	**Year 0 to <1 (n=108)**	**Year 1 to <2 (n=87)**	**Year 2 to <3 (n=72)**	**Year 3 to <4 (n=60)**	**Year 4 to <5 (n=54)**	**Total Study Duration Cases Per 100 Patient-Years, Cases/100 Patient Years**
Acute kidney injury	2 (1.8)	3 (3.4)	0 (0)	0 (0)	2 (3.7)	1.9
Chest pain	1 (0.9)	1 (1.1)	1 (1.4)	1 (1.7)	0 (0)	1.1
Syncope	2 (1.8)	0 (0)	0 (0)	1 (1.7)	0 (0)	0.8
Atrial fibrillation	0 (0)	0 (0)	1 (1.4)	1 (1.7)	0 (0)	0.5
Coronavirus test positive	0 (0)	0 (0)	0 (0)	0 (0)	2 (3.7)	0.5
Fluid overload	1 (0.9)	0 (0)	0 (0)	0 (0)	1 (1.9)	0.5
Hyperkalemia	1 (0.9)	1 (1.1)	0 (0)	0 (0)	0 (0)	0.5
Pneumonia	1 (0.9)	1 (1.1)	0 (0)	0 (0)	0 (0)	0.5

Note: Data are given as n (%).
Abbreviations: TEAE, treatment-emergent adverse event.
